# Microstructure, Mechanical and Tribological Properties of Si_3_N_4_/Mo-Laminated Composites

**DOI:** 10.3390/ma15082772

**Published:** 2022-04-09

**Authors:** Huaqiang Li, Wei Chen, Ziqiang Zhao, Zhaoxun Wang, Chen Zhang, Jinghui Gao, Lisheng Zhong

**Affiliations:** 1 State Key Laboratory of Electrical Insulation and Power Equipment, Xi’an Jiaotong University, Xi’an 710049, China; lhqxjtu@xjtu.edu.cn (H.L.); zc.zgp.gy@sust.edu.cn (C.Z.); gaojinghui@xjtu.edu.cn (J.G.); 2 College of Mechanical and Electrical Engineering, Shaanxi University of Science & Technology, Xi’an 710021, China; 1905008@sust.edu.cn (Z.Z.); 200512104@sust.edu.cn (Z.W.)

**Keywords:** ceramic composite, multilayer structure, mechanical characteristics, tribology

## Abstract

(1) Background: the applications of ceramic materials in a friction pair and a moving pair are limited, just because of their poor toughness and unsatisfactory tribological characteristics. In view of this, Mo as a soft metal layer was added into a Si_3_N_4_ matrix to improve its toughness and tribological characteristics. (2) Methods: The microstructure and metal/ceramic transition layer were examined using X-ray diffraction, scanning electron microscope, electron dispersive X-ray spectroscopy, and Vickers hardness. Bending strength and fracture toughness were also measured. Tribological characteristics were obtained on the pin-on-disc wear tester. (3) Results: It can be found that the multilayer structure could improve the fracture toughness of laminated composite compared with single-phase Si_3_N_4_, but the bending strength was significantly reduced. Through microstructure observation, the transition layer of Si_3_N_4_/Mo-laminated composite was revealed as follows: Si_3_N_4_→MoSi_2_→Mo_5_Si_3_→Mo_3_Si→Mo. Moreover, the addition of the Mo interface to silicon nitride ceramic could not significantly improve the tribological properties of Si_3_N_4_ ceramic against titanium alloy in seawater, and the friction coefficients and wear rates of the sliding pairs increased with the increase in load. (4) Conclusions: The process failed to simultaneously improve the comprehensive mechanical properties and tribological performance of Si_3_N_4_ ceramic by adding Mo as the soft interfacial layer. However, the utilization of metal interfacial layers to enhance the toughness of ceramics was further recognized and has potential significance for the optimization of ceramic formulation.

## 1. Introduction

Ceramic materials have high hardness, high strength, good chemical stability and excellent corrosion/wear resistance, and they have been applied in the manufacturing industry, building trades and even in the medical domain [1,2,3,4,5]. At present, ceramic materials have been used in various industrial fields, such as brake pads, radome, engines and cutting tools [6,7,8,9]. However, further applications of monolithic ceramic materials are limited due to their low resistance to fracture. Laminated composites are one of the main ways for improving the brittleness of ceramics, and are being paid more and more attention from a biomimetic point of view.

Since the 1970s, scholars have discovered that ceramic materials can be improved by bionic structural design, e.g., laminated nacre shell [10,11,12,13,14]. Zuo prepared Al_2_O_3_/Ni-laminated composites via hot-press sintering at 25 MPa under an argon atmosphere at 1400 °C for 1 h, and found that the ceramic/metal composites exhibited a higher fracture toughness of 16.10 MPa·m^1/2^ and a higher strength of 417.41 MPa than those of monolithic Al_2_O_3_ [15]. Laminated Ti/Al_2_O_3_ composite was fabricated via tap-casting and hot-press sintering a5 temperature of 1500 °C and at a pressure of 30 MPa for 1.5 h by Wu, and the results showed that the flexural strength and fracture toughness reached 361 MPa and 9.72 MPa·m^1/2^, respectively [16]. Meanwhile, Wu fabricated the laminated Ti/B_4_C composite via vacuum hot-pressing apparatus at 1800 °C under 30 MPa for 90 min. The fracture toughness of the composite reached 9.22 MPa·m^1/2^ (which increased approximately 201% compared with monolithic composite) [17]. The ceramic/metal composites, as one of the laminated composites, combines the advantages of ceramics and metals. As the crack extends into the metal interlayer, it can be deflected and bridged, due to the ductility of the metal and the residual stress from the mismatch of the thermal expansion of ceramic and metal [18,19,20,21].

Scholars also paid attention to the tribological characteristics of the laminated ceramic composites for potential application as a friction pair material [22,23]. Song et al. [24] studied the tribological performance of Al_2_O_3_/MoS_2_-BaSO_4_-laminated composites by a standard SRV friction and wear tester with reciprocating motion, and the results showed the friction coefficient of laminated composite was lower by around 0.24–0.44 times compared to monolithic alumina ceramics under a load of 70 N from RT to 800 °C, due to the combined action of MoS_2_, BaSO_4_, BaMoO_4_ and reacted products MoO_3_. Meanwhile, Tarlazzi et al. [25] investigated the tribological behaviors of Al_2_O_3_/ZrO_2_-ZrO_2_-laminated composites by using a pin-on-disc method under four different loads (10–75 N) and three sliding speeds (0.05–0.50 m/s), but the results showed that the wear resistance was not improved in the laminated structure. Hadad et al. [26] investigated the tribological performance of the multi-layer Si_3_N_4_-TiN-laminated composites at 1.3 GPa of Herzian pressure, stroke length of 2 mm, and reciprocating frequency of 10 H under unlubricated conditions, and found that the multi-layer Si_3_N_4_-TiN-laminated composites did not show a decrease in friction coefficient (around 1.2) compared to Si_3_N_4_-TiN bulk composites (around 1.1). Our research team has also been devoting efforts towards the improvement of mechanical and tribological properties of Si_3_N_4_-based ceramics for nearly ten years [27,28,29,30,31]. However, until now, we have not still found an efficient way to resolve these problems. Previously, hBN was added into the Si_3_N_4_ matrix as a second phase to improve its tribological properties. However, the incorporation of hBN significantly reduced the mechanical properties of Si_3_N_4_ ceramics due to the inert interface between the hBN and Si_3_N_4_ matrix, though the friction and wear behaviors of ceramic composites were improved. As described above, the question remains as to how to simultaneously improve the toughness and tribological properties of ceramics by laminated structural design.

Based on these factors, how to simultaneously improve the strength, toughness and tribological performance of ceramics is still a problem. In this study, the metal Mo was selected as the weak surface layer for silicon nitride-based laminated composites in the first instance because of the good self-lubricating properties of molybdenum oxides [32,33]. One novel composite material with laminar structure was fabricated in order to further enhance the mechanical and tribological characteristics while expanding the application of silicon nitride ceramics. The Si_3_N_4_/Mo-layered composites were prepared by hot-press sintering in this study. The microstructure, mechanical properties and tribological properties were systematically investigated, and a variety of test and analysis methods were adopted to present the phase composition, microstructure morphology, fracture morphology, and worn surface morphologies. Then, we revealed the mechanism behind the mechanical and tribological properties.

## 2. Experimental

### 2.1. Materials

**Figure 1:**
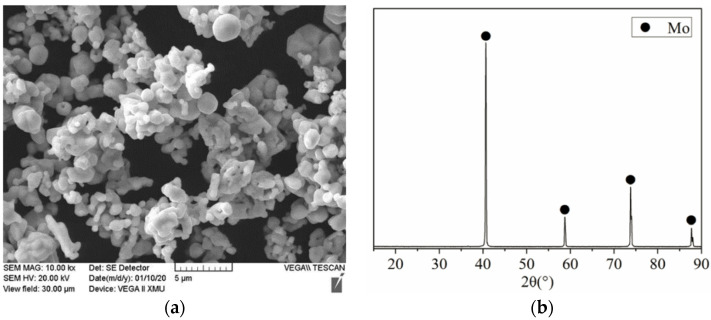
Micromorphology (**a**) and XRD result (**b**) of Mo powder.

In this study, commercial α-phase silicon nitride powder (with a purity of 99.9%, average particle size: 1.5 μm, Hefei Aijia New Material Co., Ltd., Hefei, China) and molybdenum powder (with a purity of 99.0%, average particle size: 1.0 μm, Sinopharm Chemical Reagent Co., Ltd., Shanghai, China) were utilized as raw powders to prepare Si_3_N_4_/Mo-layered composites. The micromorphology and XRD results of Mo starting powder are shown in Figure 1. In addition, Y_2_O_3_ powder (with a purity of 99%, average particle size: 0.37 μm, Aijia New Material Science & Technology Ltd., Hefei, China) and Al_2_O_3_ powder (with a purity of 99.9%, average particle size: 1.17 μm, Aijia New Material Science & Technology Ltd., Hefei, China) were also adopted as sintering aids.

**Figure 2:**
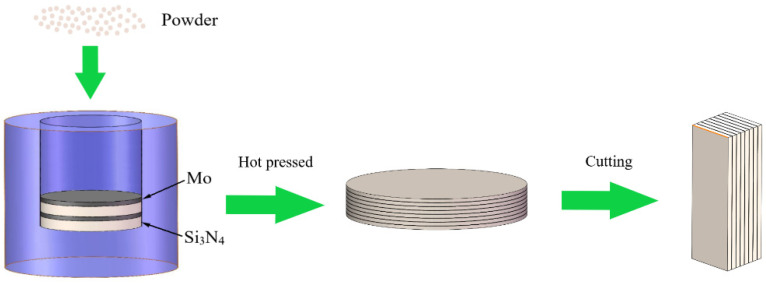
The schematic diagram of fabrication procedure for the laminated samples.

Aiming to realize the uniform distribution of Mo layer, the silicon nitride matrix composite with two-layer thickness ratio (11:1 and 9:1) of Si_3_N_4_/Mo was designed under the conditions of a certain sample thickness according to the relevant reference [34]. The laminated structure of Si_3_N_4_/Mo composite was firstly designed as shown in Figure 2, and the design parameters (e.g., layer number, thickness ratio and Mo mass ratio) is shown in Table 1.

Then, the Si_3_N_4_ powders with 4% Y_2_O_3_ and 6% Al_2_O_3_ powders were ball-milled using zirconia oxide balls for 5 h at 100 rpm in alcohol, and then the mixed powders were constantly stirred and dried in a drying oven. Subsequently, the dried powders of mixed-ceramics and Mo powder were weighted according to the relevant design parameters. Next, the ceramic and Mo powders were successively stacked in layers in a stainless-steel mold, and the slab of multilayer sample was cold pressed for 10 min at a pressure of 30 MPa. Finally, the multilayer slab was hot-pressed sintered for 30 min at a pressure of 30 MPa and a temperature of 1800 °C in a nitrogen atmosphere. In this way, a Si_3_N_4_/Mo composite disc with a size of Φ 45 mm × 6 mm was prepared, and a pure Si_3_N_4_ disc was also prepared as reference for comparison with the mechanical properties of layered composite. Additionally, then, the test piece with a size of 35 mm × 3 mm × 4 mm was cut from the disc sample for its physical and mechanical properties, and the test piece with a size of 10 mm × 5 mm × 5 mm was also cut for tribological properties as shown in Figure 2.

### 2.2. Test Procedure

To obtain the physical and mechanical properties of a laminated ceramic composite, the density and porosity of the ceramic composite were measured according to the Archimedes methods, the bending strength of composite was determined by a three-point bending test with a span length of 30 mm and a crosshead speed of 0.5 mm/min. The Vickers hardness was measured on polished surface with a load of 10 N for 15 s, and each sample has at least 10 Vickers indentations on its surface. The indentation toughness is calculated by the redial crack length and the indentation diagonal length.

To obtain the friction coefficient and wear rate of the laminated ceramic composite, the tribological test of composite sliding against TC4 in artificial seawater was conducted with a pin-on-disc tribometer. In this test tribometer, an upper pin contacts a stationary disc. The pin specimen (11SM and 9SM in Table 1) with a filleted square end was used to form flat contacts; the disc, as the mating materials, was machined from TC4, in a size of 44 mm in diameter and 5 mm in thickness. The TC4 disc was finished by grinding to achieve a surface roughness (Ra) of about 0.1 μm, and the laminated composite was carefully polished to a surface roughness of Ra 0.1–0.3 μm. The pin and disc samples were both ultrasonically cleaned in fresh alcohol. The discs were fixed, and the composite pin was rotated at a speed of 500 r/min (0.836 m/s) and normal loads of 10 N (0.4 MPa), 20 N (0.8 MPa) and 30 N (1.2 MPa). Meanwhile, the total sliding time was set as 20 min. Additionally, the liquid medium artificial seawater prepared according to Standard ASTM D 1141-98 (as shown in Table 2). The initial running-in period was not accounted for the calculation of friction coefficient (*f*) and wear rate (*w*). The friction coefficient is directly determined by the tester. Additionally, the wear rate is defined by *w* = Δ*m*/(*ρPL*), where Δ*m* represents the mass wear volume assessed by weight loss using a microbalance (accuracy = 0.1 mg), *P* is the normal load, *L* is the sliding distance, and *ρ* is the density. Friction coefficients and wear rates were obtained from the average of the values taken from three runs.

**Figure 3:**
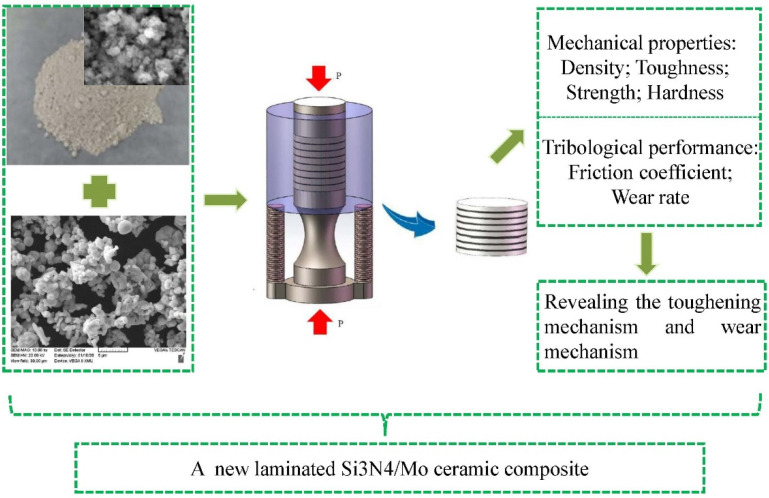
Roadmap of this research work.

The composite samples were deeply etched in a solution of NaOH for 2 min, and the microstructure of ceramic composite was observed by scanning electron microscope (SEM). Additionally, the phase composition of composite was analysed by X-ray diffract meter (XRD). The morphological analysis and chemical characterization of the wear surfaces were made by SEM/EDS. In this case, the toughening mechanism and wear mechanism of Si_3_N_4_/Mo composite was revealed in this study, as shown in Figure 3.

## 3. Results and Discussion

In this study, one new laminated material—the Si_3_N_4_/Mo composite—was developed to improve the mechanical and tribological properties of silicon nitride ceramic. Due to the dependence of material performance on the microstructure of materials, the phase composition and microstructure was firstly analyzed in this section. Then, the mechanical properties of the composite material were also analyzed, and the underling toughening mechanism was also discussed. Meanwhile, the tribological performance was tested, and the wear mechanism is analyzed in depth in the folowing subsection.

### 3.1. Phase Composition and Microstructure

**Figure 4:**
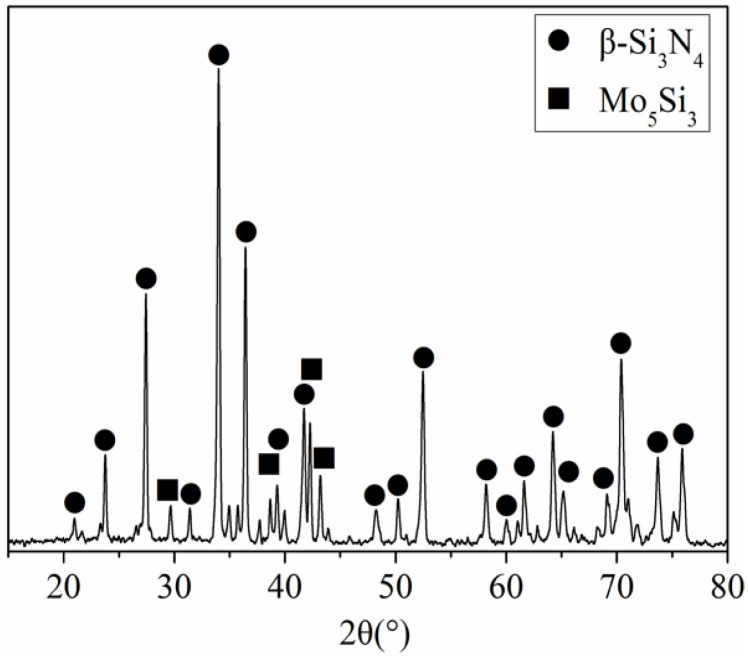
The XRD result of 9SM composites.

It is well known that α-phase silicon nitride starting powder changed into β-phase silicon nitride bulk during the sintering process. In this study, we must reveal the influence of Mo powder on Si_3_N_4_ phase transformation and the existence form of Mo powder in the ceramic composite. The phase composition of Si_3_N_4_/Mo-laminated composite was analyzed by XRD, and the result is shown in Figure 4. It can be seen that the laminated composite is composed of β-Si_3_N_4_ and Mo_5_Si_3_ phases were detected on the surface of 9SM composite. Obviously, α-Si_3_N_4_ was completely transited to β-Si_3_N_4_ during the sintering process. The other main phase Mo_5_Si_3_ is one molybdenum–silicon compound with a certain brittleness, and this compound should be a reaction product between Si_3_N_4_ and Mo during the fabrication process. No Mo was detected on the composite surface, and the Mo layer on the surface should react with silicon nitride to some compounds (e.g., Mo_5_Si_3_).

**Figure 5:**
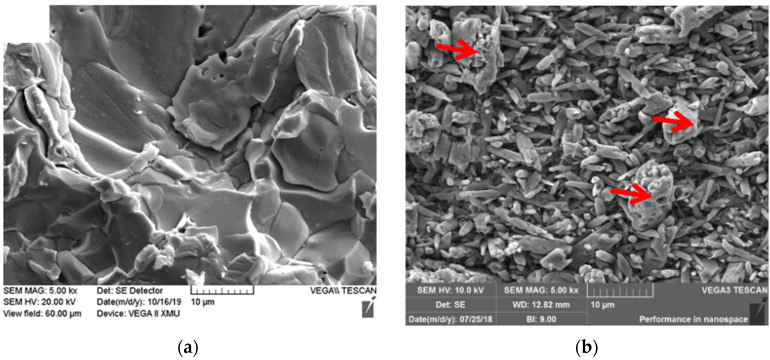
Microstructure of metal layer (**a**) and ceramic matrix (**b**) in 9SM-laminated composites.

Figure 5 shows the micromorphology of metal layer and ceramic matrix for 9SM-laminated composites. Figure 5a shows the microstructure of the metal layer, and it can be seen that some cracks appear in the metal layer, and the characteristics of brittle phase are very obvious. Figure 5b shows the microstructure of silicon nitride matrix, and it can be clearly seen that the ceramic matrix is mainly composed of columnar crystal, and some special compounds (indicated by the red arrows) are distributed sporadically on the matrix.

**Figure 6:**
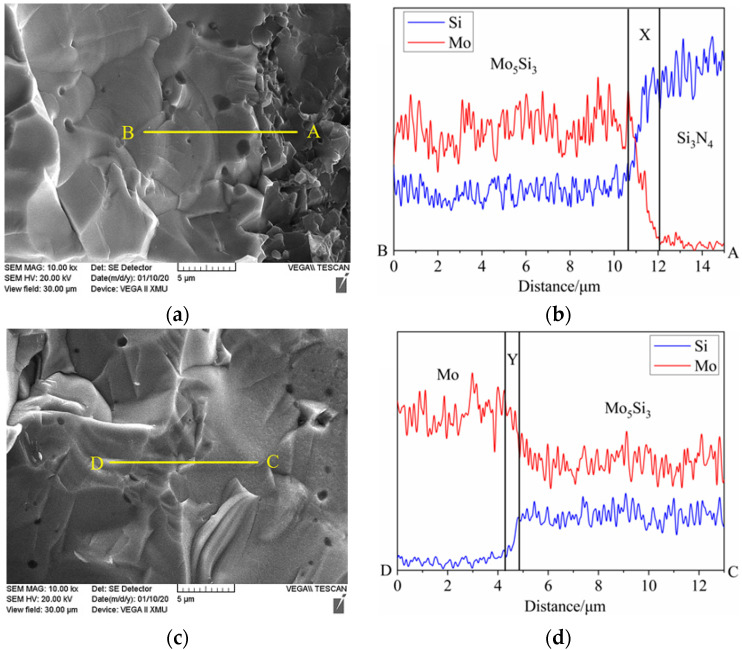
Enlarged morphologies of the interface area near ceramic matrix (**a**) and the corresponding EDS line scan result (**b**); enlarged morphology of the interface area near metal layer (**c**) and the corresponding EDS line scan result (**d**) for 9SM composite.

Figure 6 gives the enlarged morphologies of interface between ceramic matrix and metal layer, and the corresponding EDS analysis results. Figure 6a shows the enlarged morphology o the interface area near the ceramic matrix (“A” area), and it can be seen that there is a transition area between ceramic matrix (“A” area) and the interface area (“B” area). Figure 6b shows the corresponding EDS analysis result from “A” to “B” area, and it can be found that Mo element gradually appears and increases to a certain value. After the transition of the “X” region, the relative ratio of Mo and Si elements reached a stable state of about 5:3. Combined with the XRD result, it can be confirmed that Mo_5_Si_3_ formed in the interface between the ceramic matrix and the Mo layer. Figure 6c shows the enlarged morphology of the interface area near the metal layer, and the corresponding EDS analysis result is shown in Figure 6d. Figure 6d presents that along the path from the interface area (“C” area) to metal layer (“D” area), the Si element gradually decreases, while the Mo element gradually increases. After passing through the “Y” area, the relative ratio with Mo element reached a stable state of about 3:5. As discussed above, it can be concluded that one of the interface compounds is Mo_5_Si_3_, and there are still other Si-Mo compounds in the interface layer.

According to the relevant studies [35], the reaction of Si_3_N_4_ and Mo can take place at high temperatures, as follows.

(1) $$3\mathrm{Mo}+2{\mathrm{Si}}_{3}N_{4}\to 3{\mathrm{MoSi}}_{2}+4N_{2}$$

(2) $$20{\mathrm{MoSi}}_{2}+48\mathrm{Mo}\to {\mathrm{Mo}}_{3}\mathrm{Si}+13{\mathrm{Mo}}_{5}{\mathrm{Si}}_{3}$$

(3) $${\mathrm{Mo}}_{5}{\mathrm{Si}}_{3}+4\mathrm{Mo}\to 3{\mathrm{Mo}}_{3}\mathrm{Si}$$

From the chemical equations above, MoSi_2_, Mo_3_Si and Mo_5_Si are all the chemical products of Si_3_N_4_ with Mo. Combined with EDS analysis results, it can be inferred that substance in region “X” (in Figure 6b) should be MoSi_2_, and the substance in region “Y” (in Figure 6d) should be Mo_3_Si. Therefore, the material distribution from the ceramic matrix to the Mo layer is: Si_3_N_4_→MoSi_2_→Mo_5_Si_3_→Mo_3_Si→Mo; namely, the transition layer between ceramic matrix and metal layer is Si_3_N_4_→MoSi_2_→Mo_5_Si_3_→Mo_3_Si→Mo.

From the discussion above, Si_3_N_4_ ceramics reacted with Mo to form molybdenum silicide. It is well known that the thermodynamic condition for these reactions is that the corresponding Gibbs free energy must be negative. Additionally, the mathematical expression for the Gibbs free energy at a given temperature T can be described below.

(4) $$\Delta G_{T}^{0}=\Delta H_{298}^{0}-T\Delta S_{298}^{0}+\int_{298}^{T}C_{p}dT-T\int_{298}^{T}\left(C_{p}/T\right)dT$$

where Δ*G* is the difference in Gibbs free energy of the chemical reaction, Δ*H* is the difference in the enthalpy of the chemical reaction, Δ*S* is the difference in the entropy of the chemical reaction, *T* is the reaction temperature (*T* = *C* + 273.15, *Kelvin*), and *C_p_* is the molar heat capacity of the substance at 298 K.

Table 3 lists the relevant enthalpy and entropy of the products and reactants. According to Formula (4), the Gibbs free energies of reaction Equations (1)–(3) are calculated to determine the possibility of the spontaneous occurrence for the chemical reactions.

The Gibbs free energy calculation results of the chemical reaction for the Si_3_N_4_/Mo layered composite are shown in Table 4. From the table, it can be seen that the Gibbs free energies of the reactions are all negative. From this, Si_3_N_4_ and Mo could undergo the chemical reactions to form MoSi_2_, Mo_5_Si_3_, and Mo_3_Si. Additionally, because the Gibbs free energy of Equation (2) is the lowest, the drive for this reaction to happen should be highest. Because of this, Mo_5_Si_3_ occupied the largest proportion in the transition layer. Overall, it can be verified that for Si_3_N_4_/Mo-laminated composite, one transition layer (Si_3_N_4_→MoSi_2_→Mo_5_Si_3_→Mo_3_Si→Mo) formed between β-phase Si_3_N_4_ matrix and Mo metal layer.

### 3.2. Mechanical and Tribological Properties

Based on the above, it can be confirmed that one chemical transition layer formed between Si_3_N_4_ and Mo. Meanwhile, the Mo-Si compounds such as Mo_5_Si_3_ present brittle characteristics (as shown in Figure 5a). Such a microstructure should affect the mechanical and tribological properties of the laminated composite.

Table 5 gives the mechanical properties of Si_3_N_4_/Mo-laminated composites. Compared with single-phase Si_3_N_4_, the composite presented lower bending strength and higher toughness. The ceramic matrix in the composite presented a similar hardness compared with single-phase Si_3_N_4_. It is obvious that the Si_3_N_4_/Mo-laminated composites do exhibit good toughness, but the strength is very poor.

The similar hardness of the ceramic matrix to the single Si_3_N_4_ would be attributed to the completed phase transformation of Si_3_N_4_ during the same sintering process (as shown in Figure 5). Meanwhile, Mo_5_Si_3_, as a reaction product, is a brittle substance between the ceramic matrix and the metal layer. This substance has a large difference in lattice constant (a/c ≈ 2), and the coefficient of thermal expansion is anisotropy (α_c_/α_a_ ≈ 2). Therefore, cracks would appear during the growth of a Mo_5_Si_3_ single crystal, and this is the reason for the large number of cracks observed in the metal layer as shown in Figure 5. Consequently, the ability of the laminated composite to resist cracking is reduced. Thus, the bending strength of laminated composite was obviously lower than that of the single-phase ceramic.

**Figure 7:**
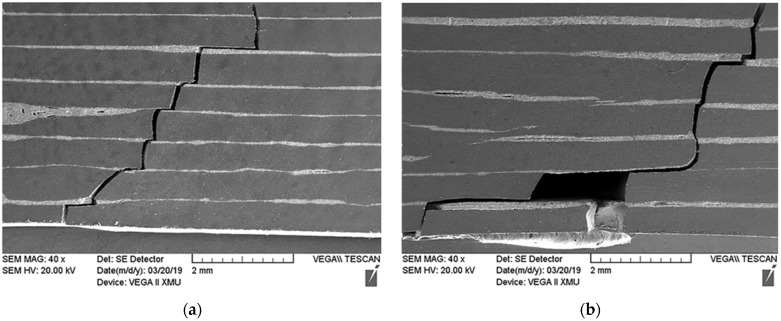
The fracture trend of Si_3_N_4_/Mo laminated ceramic composites: (**a**) 9SM; (**b**) 11SM.

On the other hand, due to the difference of thermal expansion coefficient between the interface layer and the matrix layer, tensile stress and compressive stress were generated at the interface between the ceramic matrix and metal layer. In this case, when the crack propagated in the laminated composite, the existence of residual stress caused the crack to deflect at the interface of the laminated composite, thus consuming the fracture energy and improving the fracture toughness of the laminated composite. Figure 7 shows the tendency of a zigzag crack propagation of 9SM and 11SM composites. Therefore, the fracture toughness of laminated composite is higher than that of single-phase ceramic. From Figure 7, it can be also seen that the crack deflects as it passes through the metal layer in 9SM composite, while some cracks directly penetrate the metal layer and the ceramic matrix in 11SM composite. Combined with Table 1, it can be concluded that, when the content of Mo in the laminated composite is higher, it is beneficial to prevent the diffusion of cracks and improve the fracture toughness of the material.

**Figure 8:**
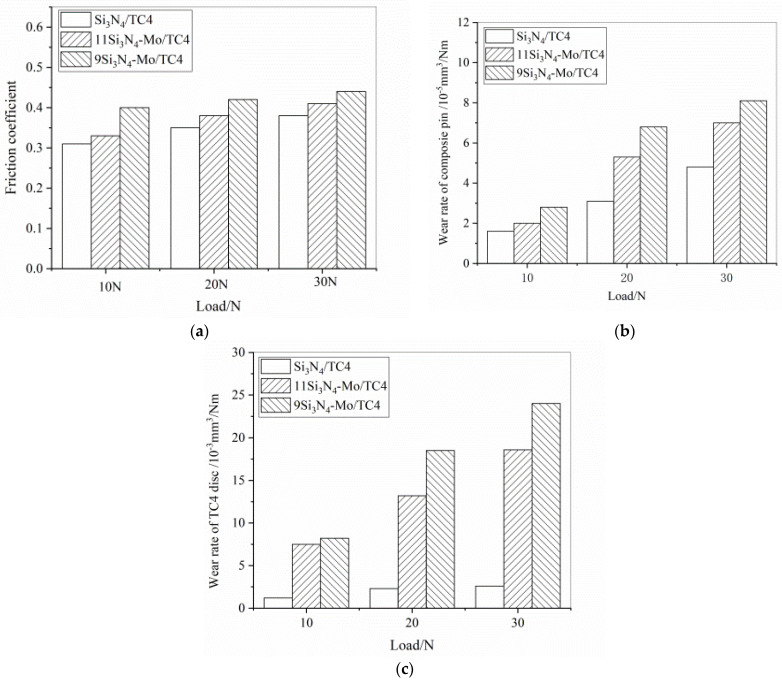
Friction coefficient (**a**), wear rate of pin (**b**) and disc (**c**) for Si_3_N_4_/Mo-laminated composite sliding against TC4 pairs in artificial seawater.

The tribological characteristics of the laminated composite were also carried out, and the friction coefficients and wear rates of Si_3_N_4_/Mo-laminated composite sliding against TC4 pairs at different load in seawater are shown in Figure 8. From this figure, it can be seen that the friction coefficients of the sliding pairs increase with the increase in load (from 10 N to 30 N) in Figure 8a. In general, the friction coefficient of laminated composite/TC4 is at a range from 0.3 to 0.5, and the friction coefficient of 9SM/TC4 pair is highest. From Figure 8b,c, the wear rates of pin and disc both increase with the increase in load. Additionally, the wear rates of laminated composite/TC4 pair are higher than the single ceramic/TC4 pair. Combined with the results of friction coefficient and wear rate, it can be seen that adding Mo as the interfacial layer to the Si_3_N_4_ ceramic matrix did not improve its tribological properties.

**Figure 9:**
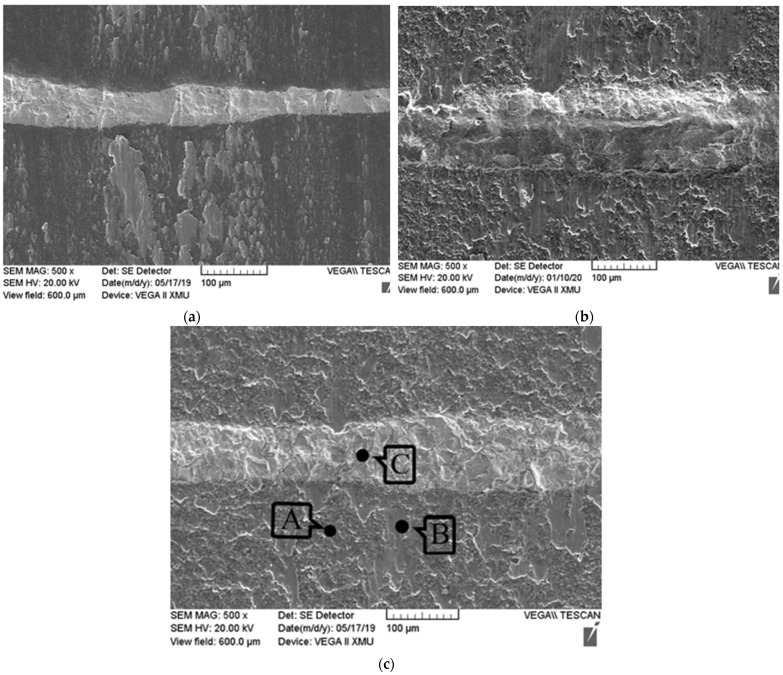
The morphologies of the worn surfaces for 11SM pin in artificial seawater under different loads: (**a**) 10 N; (**b**) 20 N; (**c**) 30 N.

**Figure 10:**
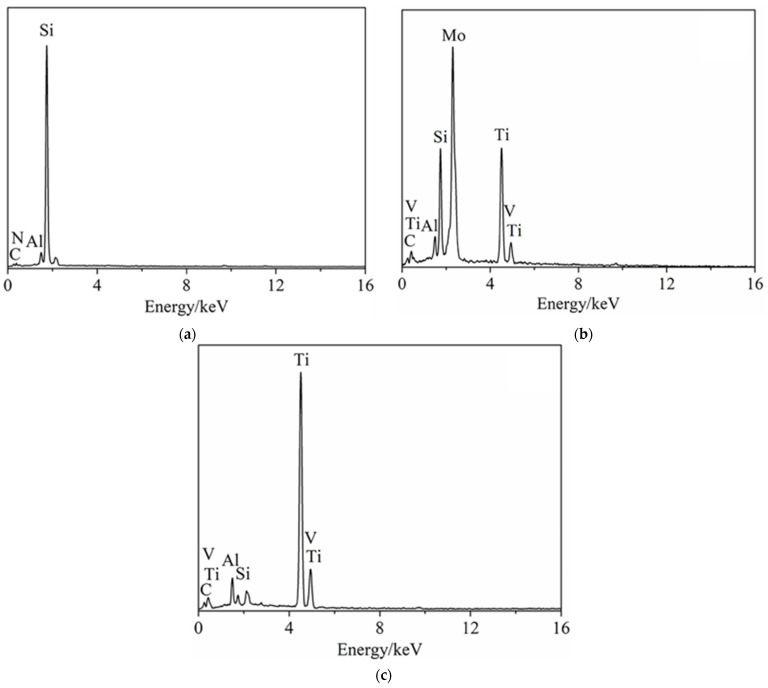
The analysis results of the friction surface of 11 Si_3_N_4_/Mo pin sample under a load of 30 N: (**a**) A zone; (**b**) B zone; (**c**) C zone in Figure 9c.

Figure 9 shows the morphologies of the worn surfaces of 11SM pins in seawater. From the figures, it can be seen that the metal transfer layer gradually appears on the worn surface of the laminated composite with the increase in load (Figure 9a–c). At loads of 20 N and 30 N, the worn surface becomes significantly coarser (Figure 9b,c). The EDS analysis results of the worn surface of 11SM pin at a load of 30 N is shown in Figure 10. It can be seen that the ceramic matrix region is mainly composed of two different regions (“A” and “B” zone in Figure 9c). The region “A” is mainly composed of Si elements, while the region “B” is mainly composed of metallic elements such as Ti, Mo and V (Figure 10a,b). Obviously, some metal transfer layers formed on the wear surface of composite pin. The wear surface of metal interface layer (“C” zone in Figure 9c) is mainly composed of Ti, V and other elements as shown in Figure 10c. It is interesting that the Mo element is not detected on the metal interface layer, which may be also attributed to the formation of metal transferred layer from TC4 disc. From the analysis results above, it can be found that even under the cooling and lubrication of seawater, obvious adhesion wear still appeared when the laminated composite ceramic was matched with the titanium alloy.

**Figure 11:**
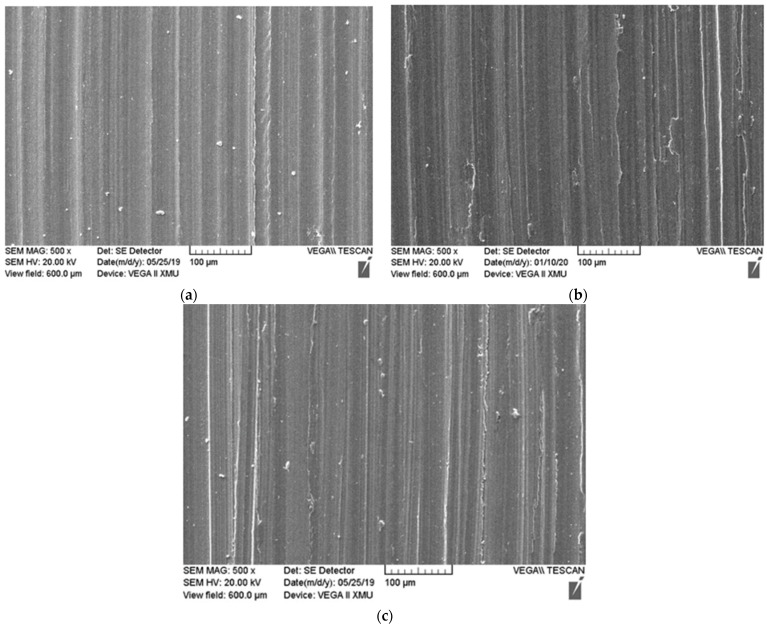
The morphologies of worn surface for TC4 against 11 Si_3_N_4_/Mo pin sample in artificial seawater under different loads: (**a**) 10 N; (**b**) 20 N and (**c**) 30 N.

The worn surfaces of the TC4 disc against the 11SM pin are shown in Figure 11. In the figure, obvious furrows can be observed on the wear surfaces of the TC4 disc. Meanwhile, when the load is 20 N and 30 N, the worn surface of TC4 disk appears the characteristics of repeated adhesion. Obviously, when the Si_3_N_4_/Mo composite slid against the TC4, the surface of the titanium alloy disc was ploughed by the micro-bulge on the surface of composite pin. Due to the incorporation of the Mo layer, more serious adhesion wear occurred for the composite/TC4 pair compared with Si_3_N_4_/TC4. Therefore, the friction coefficient and wear rate of the Si_3_N_4_/Mo against the TC4 were both higher than those of the Si_3_N_4_ against the TC4.

Based on the discussion above, when the Mo was added into the Si_3_N_4_ matrix as an interface layer, the fracture toughness of the ceramics was slightly enhanced to 11.2 MPa·m^1/2^, but the strength was reduced to 330 MPa. K. Balazsi [36] prepared a layered silicon nitride–zirconia composite with MLG with a fracture toughness of 4.6 MPa·m^1/2^, and a bending strength of 264 MPa. Sun Mengyong [37] also indicated that a SiC/BN composite presented a higher fracture toughness of 8.5 MPa·m^1/2^ and a lower bending strength of 300 MPa. Compared with the experimental data, the mechanical properties of the Si_3_N_4_/Mo-laminated composite are better, especially for the high fracture toughness.

However, even in seawater, the tribological properties of Si_3_N_4_ against the titanium alloy could not be effectively improved. On the contrary, the incorporation of the Mo interface layer played a role in the degradation.

It can be seen from the research work in this paper that the Si_3_N_4_/Mo composite materials have lower strength and poor tribological properties, so they are not suitable as a friction pair material. However, the composite materials also have higher toughness and moderate hardness, and the addition of the metal Mo should improve the thermal conductivity of the silicon nitride material; therefore, these composite materials can be considered for use in a radome and other non-stressed parts.

## 4. Conclusions

In this study, Mo was added into a silicon nitride matrix as a metal interface layer, and then the Si_3_N_4_/Mo-laminated ceramic composite was successfully fabricated via hot-press sintering. The mechanical and tribological properties were investigated in this study, and the following conclusions were obtained.

(1) The transition layer from the ceramic matrix to Mo interface layer was Si_3_N_4_→MoSi_2_→Mo_5_Si_3_→Mo_3_Si→Mo. Meanwhile, the compounds including MoSi_2_, Mo_5_Si_3_ and Mo_3_Si were all the products of the reaction between the ceramic matrix and the metal interface layer. The transition layer was mainly composed of brittle phase Mo_5_Si_3_, which had a negative effect on the mechanical properties of laminated composite.
(2) The ductility of the metal Mo layer and the residual stress between the ceramic matrix and the metal layer resulted in crack deflection and branching, as well as a higher fracture toughness for the laminated ceramic composite.
(3) The incorporation of the Mo interface layer to the silicon nitride matrix degraded the tribological properties of the Si_3_N_4_ ceramic sliding against the TC4 in seawater. Meanwhile, with the increase in load, the friction coefficients and wear rates both also increased.

In general, the addition of the Mo metal as an interface layer to the Si_3_N_4_ ceramic matrix was intended to improve the mechanical and tribological properties of ceramics, which failed. However, this research also suggests that adding a metal as an interface layer to a ceramic matrix does indeed toughen the ceramic material. Hence, the authors of this paper indicate that the composite can be considered for use in a radome and other non-stressed parts.

## Figures and Tables

**Table 1:** Composition of the Si_3_N_4_/Mo composites and their numbers.

Number	Thickness Ratio of Si_3_N_4_ to Mo	Layer Number of Mo Layer	Molybdenum Mass Ratio (%)
11SM	11:1	7	24.7
9SM	9:1	7	30.2

**Table 2:** Chemical composition of artificial seawater.

Constituent	NaCl	Na_2_SO_4_	MgCl_2_	CaCl_2_	SrCl_2_	KCl	NaHCO_3_	KBr	H_3_BO_3_	NaF
Concentration (g·L^−1^)	24.53	4.09	5.20	1.16	0.025	0.695	0.201	0.101	0.027	0.003

**Table 3:** Enthalpy and entropy of chemical reactants.

Materials	*S* (J·K^−1^·mol^−1^)	*H* (KJ·mol^−1^)	*C_p_* (J·K^−1^·mol^−1^)
Mo	23.96	0	22.93 + 5.44 × 10^−3^ T
Si_3_N_4_	112.97	−744.75	76.34 + 109.04 × 10^−3^ T − 6.54 × 105 T^−2^
MoSi_2_	65.02	−131.71	67.83 + 11.97 × 10^−3^ T − 6.57 × 105 T^−2^
Mo_5_Si_3_	207.34	−309.62	183.36 + 35.04 × 10^−3^ T − 12.00 × 105 T^−2^
Mo_3_Si	106.15	−116.40	85.84 + 22.68 × 10^−3^ T − 0.32 × 105 T^−2^
N_2_	191.5	0	27.86 + 4.27 × 10^−3^ T

**Table 4:** Gibbs free energy of friction chemical reaction.

Chemical Equation	$\Delta G_{2098\;K}\;(\mathrm{kJ}\cdot {\mathrm{mol}}^{-1})$
$3\mathrm{Mo}+2{\mathrm{Si}}_{3}N_{4}\to 3{\mathrm{MoSi}}_{2}+4N_{2}$	−255.21
$20{\mathrm{MoSi}}_{2}+48\mathrm{Mo}\to {\mathrm{Mo}}_{3}\mathrm{Si}+13{\mathrm{Mo}}_{5}{\mathrm{Si}}_{3}$	−1873.38
${\mathrm{Mo}}_{5}{\mathrm{Si}}_{3}+4\mathrm{Mo}\to 3{\mathrm{Mo}}_{3}\mathrm{Si}$	−21.54

**Table 5:** Mechanical properties of composite materials.

Properties	Si_3_N_4_	11SM	9SM
Vicker’s hardness (GPa)	14.9	14.0 (ceramic matrix)	14.6 (ceramic matrix)
Bending strength (MPa)	818	275	330
Fracture toughness (MPa·m^1/2^)	8.50	10.7	11.2

## Data Availability

Not applicable.
